# Correlation between lifestyle patterns and overweight and obesity among Chinese adolescents

**DOI:** 10.3389/fpubh.2022.1027565

**Published:** 2022-11-03

**Authors:** Yuanyuan Ma, Huipan Wu, Jinbo Shen, Jian Wang, Jinxian Wang, Yuxin Hou

**Affiliations:** ^1^Research Center for Health Promotion of Children and Adolescents, Taiyuan Institute of Technology, Taiyuan, China; ^2^Department of Physical Education, Shanxi University, Taiyuan, China

**Keywords:** overweight and obesity, lifestyle, dangerous behavior, physical exercise, adolescent

## Abstract

Lifestyles such as physical exercise, sedentary behavior, eating habits, and sleep duration are all associated with adolescent overweight and obesity. The purpose of this study was to investigate how Chinese adolescents' lifestyles clustered into different lifestyle patterns, and to analyze the correlation between these patterns and adolescent overweight and obesity. The investigated respondents included 13,670 adolescents aged 13–18 from various administrative regions in China. Latent class analysis was employed to cluster the lifestyles of adolescents, χ2 test and Logistic regression were used to explore the relationship between lifestyle patterns and overweight and obesity in adolescents. The results identified 6 types of Chinese adolescents' lifestyle patterns, as well as the significant differences in gender and age. The adolescents with high exercise-high calorie diet had the lowest risk of overweight and obesity, and the adolescents with low consciousness-low physical activity and low consciousness-unhealthy had the highest risk of overweight and obesity, which were 1.432 times and 1.346 times higher than those with high exercise-high calorie diet, respectively. The studied demonstrated that there was a coexistence of healthy behaviors and health-risk behaviors in the lifestyle clustering of Chinese adolescents. Low physical exercise and high intake of snacks and carbonated beverages were the most common. Physical exercise and health consciousness were the protective factors of overweight and obesity in adolescents.

## Introduction

In the recent decades, overweight and obesity have been highly prevalent among children and adolescents populations, and moreover, it has proved to be a major challenge of global health (1). The related data indicated that it was expected that the global obesity of children and adolescents would increase by 60% in the future 10 years, and the total number would reach 250million by 2030, which would undoubtedly put great pressure on the world public health system (2). With the improvement of social development and economic level, the lifestyle and dietary pattern of Chinese adolescents have undergone tremendous changes (3, 4). In the recent 40 years, the height and weight of Chinese teenagers has presented an upward trend, at the same time, the overweight and obesity rate of teenagers has also continued to rise. According to Report on Nutrition and Chronic Diseases of Chinese Residents (5), the overweight and obesity rate of children and adolescents aged 6 to 17 in China has reached 19%, and the growth rate of children's overweight and obesity from 2002 to 2012 was 3.44 times that of adults. Obesity has seriously affected the physical and mental health development of children and adolescents (6).

Overweight and obesity in adolescence would significantly increase the morbidity rate of metabolic disorders, insulin resistance and diabetes, cardiovascular disease, nonalcoholic steatohepatitis, musculoskeletal and psychological disorders. In the meanwhile, it was significantly associated with the health status in their adulthood periods (7–10). In view of the complexity and long-term properties of overweight and obesity in the process of occurrence and development, as well as the immaturity of adolescents' physical and mental development, improving lifestyle-related behaviors and environments has been a scientific method characteristics with safety and economy (11). Under the described background, many studies have been conducted in the aspect of the interaction between overweight and obesity with a variety of lifestyle factors such as physical exercise, sedentary behavior, eating habits and sleep duration. The results illustrated that lifestyle had a direct impact on adolescent overweight and obesity (12, 13). In addition, health-risk behaviors are not isolated as a major contributor to overweight obesity in adolescents, and these behaviors may gradually stabilize or increase further in adulthood (14). Therefore, assessing the lifestyle characteristics of adolescents can identify important factors that contribute to obesity, thereby determining their risk of overweight obesity and developing interventions. In addition, it can also provide a reference for determining the clinical manifestations of cardiometabolic diseases and the clinical manifestations in the subclinical inflammatory process.

In the current status, limited to objective conditions and reality, most studies put more emphasis on the impact of a single behavioral factor on overweight and obesity in adolescents. Although the related factors have been controlled, the explanatory power for complicated interactions was limited (15). It has been demonstrated that when a single negative health behavior and various positive health behaviors occurred simultaneously, lifestyle behaviors were not necessarily associated with negative health outcomes (16). In fact, human behavior is inherently multivariate and interactive. For example, physical activity, sedentary behaviors, and eating habits may behave in more complex ways, with cumulative effects on obesity development in adolescents (17). In this regard, cluster analysis and other “individual-centered” research paradigms were employed to analyze the lifestyles of adolescents, so as to restore the daily lifestyle more comprehensively. Current research has focused on clustering analysis of physical activity, sedentary behavior and eating behavior in adolescents. Other components of lifestyle, such as sleep habits and health awareness, are also significant variables affecting adolescent obesity, so future research should be analyzed from a multifactorial perspective of adolescent lifestyle behavior. In addition, the vast majority of such surveys were conducted in high-income countries, with a lack of research in middle- and low-income countries (18). The lifestyle patterns that lead to adolescent obesity are more complex, and are significantly related to factors such as race, culture, and social psychology, and more research support is still needed.

In the present paper, the lifestyles of adolescents such as physical exercise, sedentary behavior, eating habits, sleep duration were taken as a whole. The subgroups of Chinese adolescents' lifestyles were analyzed through latency class analysis to understand the accumulation of dangerous health behaviors in their lifestyles. Moreover, the differences of overweight and obesity among the adolescents with different lifestyle patterns were compared and analyzed to explore the correlation between lifestyle patterns and overweight and obesity. The present work aims to provide a theoretical basis for the individualized intervention of overweight and obesity, so as to effectively reduce the risk of overweight and obesity among the adolescents.

## Methods

### Study design and participants

According to the six administrative regions (East China, North China, Central South China, Northwest China, Southwest China, and Northeast China) regulated by Chinese traditional administrative regions, about 200 male and 200 female students in each grade of junior high school were selected by the class cluster sampling method, following the ratio of about 1:1 for men and women, urban and rural areas, and north and south regions. From September to November 2019, in the cities of Nanjing, Datong, Wuhan, Lanzhou, Kunming, and Changchun, respectively, through the Adolescent Living Habits Questionnaire, the basic information and lifestyles of nearly 15,000 children and adolescents in China were collected. Eventually, a total of 13,670 valid data were obtained, including 6,911 boys and 6,759 girls aged from 13 to 18 years.

Before starting the investigation, the research group conducted unified training on the specific requirements of the test for the participating investigators. In the survey, the investigators used the time of the class meeting class or self-study class, accompanied by the class teacher or the classroom teacher, to explain the questionnaire content, fill in the precautions and answer questions on the spot. Students fill out the questionnaire themselves after obtaining informed consent. Investigators distributed and collected questionnaires on the spot, and eliminated questionnaires with logical contradictions.

### BMI and lifestyle related behavior measures

1) Classification criteria of body mass index (BMI). During the test, the participate is required to wear barefoot and single clothes and pants on the instrument, and measure the height and weight of children and adolescents in accordance with the requirements of physical health standards (19). BMI = weight (kg)/[height (m)]^2^, and the classification standard is in accordance with the WHO standard (20): ≥1 s to 2 s represents overweight, and >2 s represents obesity.
2) Health consciousness (21): Emphasis on health and physical exercise consciousness are ordinal level variables with 5 categories. According to the respondents' answers, we assign 1 to “not important,” 2 to “generally important,” 3 to “relatively important,” 4 to “important,” 5 to “very important.” “Very important” and “important” are defined as healthy, while the others are defined as unhealthy.
3) Physical activity (22, 23): The Physical Activity Level Assessment Questionnaire for children and adolescents aged 7 to 18 years in China was used to investigate the PA status. Exercise time survey: mainly investigated the amount of time testers engaged in physical activity per day, classified as healthy (>60 m/d) or unhealthy (≤60 m/d). Video Screen Time Survey: The survey mainly investigated the amount of time testers spent watching TV, playing games and browsing mobile phones per day, classified as healthy (<2 h/d) defined or unhealthy (≥2 h/d). Mode of travel to and from school survey: The survey mainly investigated the mode taken by the testers to travel to and from school each day, defining regular walking and cycling as healthy and others as unhealthy.
4) Sleep duration (24): The actual daily sleep duration was used to assess the sleep duration of adolescents, which was classified as healthy (≥6 h/d) or unhealthy definite (<6 h/d).
5) Eating behavior (25): The frequency of breakfast, snacks and carbonated beverages was investigated, with “mostly eat/drink” being ≥4 times per week, “occasionally eat/drink” being 2–3 times per week, and “hardly eat/drink” being ≤1 time per week. Breakfast intake≥4 times/week is defined as healthy, and <4 times/week is defined as unhealthy. Snack intake <2 times/week is defined as healthy, and ≥2 times/week is defined as unhealthy. Carbonated beverage intake <2 times/week is defined as healthy, and ≥2 times/week is defined as unhealthy.

### Statistical analysis

Firstly, Mplus 8.3 software was used to cluster 10 variables related to lifestyle by latent class analysis, and the lifestyle pattern was determined according to the model fitting index. A stepwise regression method was used to fit the latent classification model. The study carried out fitting from the potential states 1–7, and the optimal model was selected based on the fitting index value of each model. The evaluation of model fitting degree was mainly based on Log (L) (Log Likelihood), AIC (Akaike information criteria), BIC (Bayesian information criteria), aBIC (Adjusted Bayesian information criteria) values, etc. The smaller the value was, the better the model fitting degree would be, in which BIC was the optimal indicator (26). On the basis of the indicators of the fitting model and the principle of model category simplicity, six categories of latent models were finally selected in this study.

Secondly, SPSS 24.0 statistical software was employed to compare the category probability of different lifestyle patterns of adolescents, and then the gender and age differences in lifestyle patterns were analyzed through multiple logistic regression analysis. Finally, chi-square test was employed to compare the detection rates of overweight and obesity among the adolescents with different lifestyle patterns, and the correlation was analyzed by binary logistic regression analysis, with the test level α = 0.05.

## Results

### Descriptive characteristic of the sample

The final study sample consisted of male (82.3%; n = 6911) and female (82.6%; n = 6759), with a mean age of 15.50 years (SD = 1.705 year), and 17.5% reported their nutritional status as “overweight and obesity”. Participant characteristics and lifestyle-related behavior are outlined in Table 1.

**Table 1:** Basic characteristics of the sample.

**Variable**	**Variable description**	**Male**	**Female**	**Overall**
Sample size	*n*	6911	6759	13670
Nutritional status	Normal	82.3%	82.6%	82.5%
	Overweight and obesity	17.7%	17.4%	17.5%
Age	Years	15.49 ± 1.708	15.50 ± 1.703	15.50 ± 1.705
School age	Junior high school	50.1%	49.8%	49.9%
	Senior high school	49.9%	50.2%	50.1%
Place of residence	Large and medium-sized cities	37.6%	35.2%	36.4%
	Small cities	37.8%	39.5%	38.7%
	Villages and towns	24.5%	25.3%	24.9%
Regions	East China	16.6%	16.8%	16.7%
	North China	16.6%	16.3%	16.4%
	South central	16.7%	16.6%	16.6%
	Northwest	16.9%	16.4%	16.6%
	Southwest	16.6%	17.2%	16.9%
	Northeast	16.7%	16.8%	16.8%
Emphasis on health	Healthy	46.8%	38.6%	42.7%
	Unhealthy	53.2%	61.4%	57.3%
Physical exercise consciousness	Healthy	82.4%	78.1%	80.3%
	Unhealthy	17.6%	21.9%	19.7%
Physical exercise time	>60 m/d	20.6%	8.6%	14.7%
	≤60 m/d	79.4%	91.4%	85.3%
Video time	<2 h/d	82.1%	85.2%	83.6%
	≥2 h/d	17.9%	14.8%	16.4%
Sleep duration	≥6 h/d	80.9%	80.2%	80.5%
	<6 h/d	19.1%	19.8%	19.5%
Way of going and leaving school	Positive	53.9%	48.5%	51.2%
	Negative	46.1%	51.5%	48.8%
Weekend leisure mode	Time outside ≥ time at home	45.6%	36.6%	41.1%
	Time outside < time at home	54.4%	63.4%	58.9%
Breakfast intake	≥4 times/week	78.4%	79.3%	78.8%
	<4 times/week	21.6%	20.7%	21.2%
Snacks intake	<2 times/week	21.7%	13.2%	17.5%
	≥2 times/week	78.3%	86.8%	82.5%
Carbonated drinks intake	<2 times/week	27.2%	36.1%	31.6%
	≥2 times/week	72.8%	63.9%	68.4%

The result in the table is percentage /% or mean ± standard deviation.

### Latent class descriptions

The study selected the optimal model based on the values of each model fitting index and the fitting results are shown in Table 2.

**Table 2:** Fitting results of lifestyle latent class analysis (LCA).

**Model**	**K**	**AIC**	**BIC**	**aBIC**	**Entropy**	**BLRT**	**LMR**
1-Class	10	150625.393	150700.623	150668.843			
2-Class	21	149144.943	149302.925	149236.89	0.381	**	**
3-Class	32	148311.419	148552.154	148450.461	0.505	**	**
4-Class	43	147914.335	148237.822	148101.172	0.488	**	*
5-Class	54	147693.925	148100.165	147928.558	0.432	**	**
6-Class	65	147548.694	148037.686	147831.122	0.490	**	**
7-Class	76	147493.559	148065.304	147823.783	0.536	**	

*Represents *P* < 0.05, **Represents *P* < 0.01.

Based on the fitting results, six categories of potential models were finally selected for this study. Each class was named according to the conditional probabilities of 10 variables of 6 classes of the latent model.

In Class 1, among the variables of the emphasis of health, physical exercise consciousness, physical exercise time, video time, weekend leisure mode, etc., the probability of negative health behavior was relatively higher. Therefore, class 1 was named as “low consciousness-low physical activity” class.

In Class 2, the subjects tended to have positive health behaviors in the probability of 9 explicit variables except physical exercise, which was named as “moderate exercise-balanced” class.

In Class 3, the subjects tended to have negative health behaviors in physical exercise, snacks and carbonated beverage intake, so they were named as “low exercise- high calorie diet” class.

In Class 4, the subjects tended to have negative health behaviors in the explicit variables, and they were named as “low consciousness-unhealthy” class.

In Class 5, the subjects tended to have negative health behaviors in the three variables related to diet, so they were named as “low diet behavior” class.

In Class 6, the positive health behaviors of the research subjects were the most obvious, physical exercise time, and the intake of snacks and carbonated beverages tended to be negative health behaviors. Therefore, they were named as “high exercise-high calorie diet” class.

**Figure 1:**
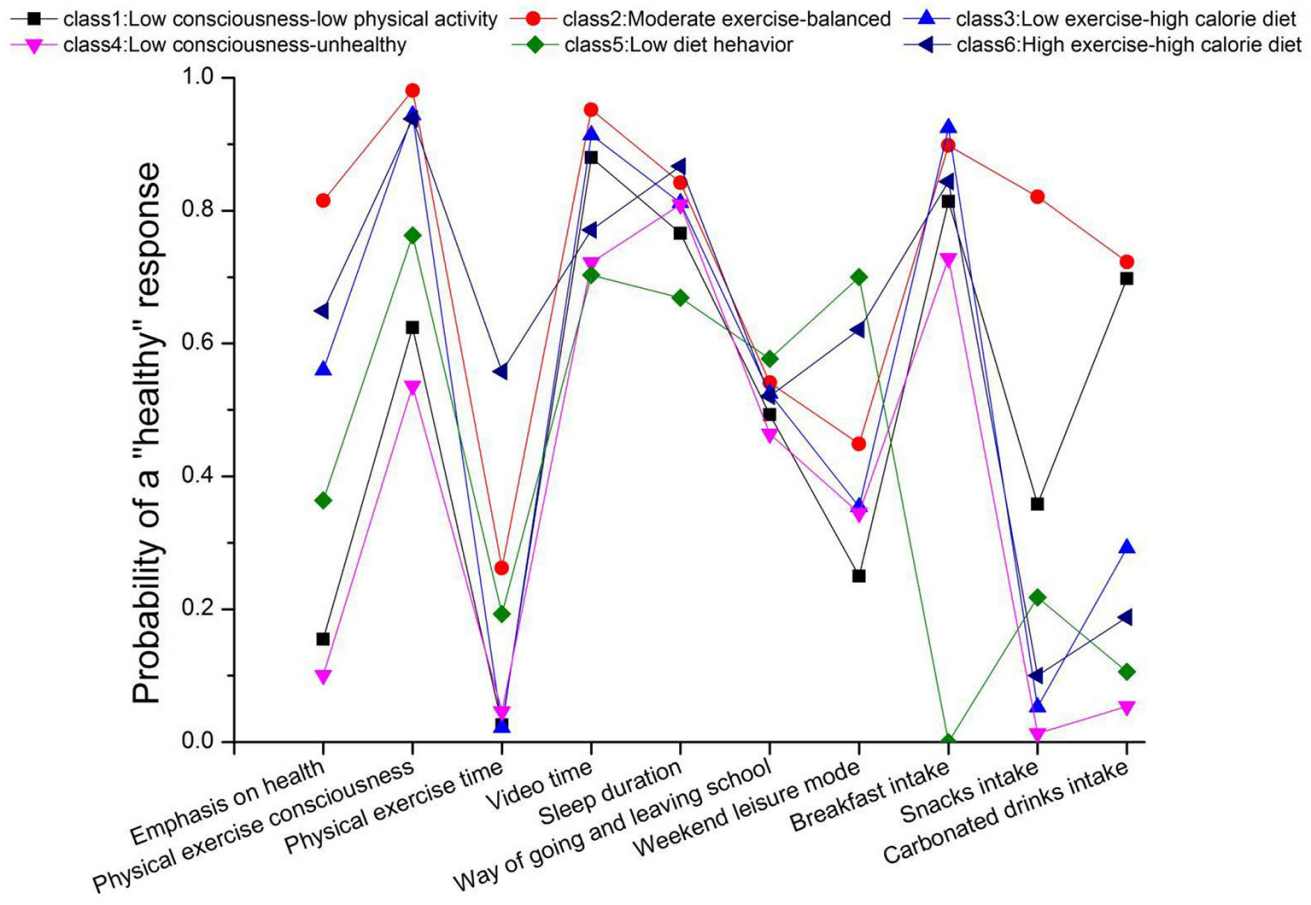
Description of the patterns of healthy lifestyle preferences. X-axis, indicators of lifestyle preferences (i.e., emphasis on health, physical exercise consciousness); y-axis, probability of a “healthy” response to each lifestyle preferences conditional on latent class membership.

The conditional probabilities of the 6 latent classes are shown in Figure 1.

### Descriptive statistics of adolescents' lifestyle patterns

The class probabilities of adolescents' lifestyle patterns were listed as follows: “low consciousness-low physical activity” class (class 1) 13.4%, “moderate exercise-balanced” class (class 2) 7.2%, “low exercise-high calorie diet” class (class 3) 43.4%, “low consciousness-unhealthy” class (class 4) 16.1%, “low diet behavior” class (class 5) 8.2% and “high exercise-high calorie diet” class (class 6) 11.8%. Then, chi square test was employed to compare the class probability of lifestyle patterns among different groups. The results indicated that there were significant differences in the class probability of lifestyle patterns in the groups with different ages and genders *(P* < 0.01).

The logistic regression analysis was carried out with gender and age as the predictive variables, and 6 lifestyle patterns as the dependent variables. The results indicated that compared with “low consciousness-low physical activity” class (class 1), female students were less possible to enter “high exercise-high calorie diet” class (class 6) (OR = 0.335, 95%CI = 0.291–0.386), “moderate exercise-balanced” class (class 2) (OR = 0.449, 95% CI = 0.383–0.527) and “low diet behavior” class (class 5) (OR = 0.616, 95% CI = 0.530–0.715). Compared with the “high exercise-high calorie diet” class (class 6), the junior high school group was less likely to enter the “low consciousness-low physical activities” class (class 1) (OR = 0.699, 95% CI = 0.608–0.803), “low exercise-high calorie diet” class (class 3) (OR = 0.743, 95% CI = 0.662–0.833), “low consciousness-unhealthy” class (class 4) (OR = 0.846, 95% CI = 0.740–0.966) and “low diet behavior” class (class 5) (OR = 0.719, 95% CI = 0.614–0.814), all of which reached significant statistical (*P* < 0.05). The results are shown in Table 3.

**Table 3:** Class probability of lifestyle patterns.

	**Class 1: Low consciousness-low physical activity type**	**Class 2: Moderate exercise-balanced type**	**Class 3: Low exercise - high calorie diet type**	**Class 4: Low consciousness - unhealthy type**	**Class 5: Low diet behavior type**	**Class 6: High exercise - high calorie diet type**
Overall	13.4%	7.2%	43.4%	16.1%	8.1%	11.8%
Gender						
Male	11.8%	9.1%	39.2%	14.4%	9.2%	16.4%
Female	15.0%	5.3%	47.7%	17.8%	7.2%	7.0%
Value of χ^2^	451.968					
Value of *P*	0.000					
OR (95% CI)	1	0.449 (0.383–0.527)	–	–	0.616 (0.530–0.715)	0.335 (0.291–0.386)
Age group						
Junior high school	12.5%	8.1%	41.6%	16.6%	7.8%	13.4%
Senior high school	14.2%	6.3%	45.2%	15.5%	8.6%	10.2%
Value of χ^2^	66.890					
Value of *P*	0.000					
OR (95% CI)	0.699 (0.608–0.803)	–	0.743 (0.662–0.833)	0.846 (0.740–0.966)	0.719 (0.614–0.814)	1

Gender is based on male students; age group is based on high school, and the results of other related variables are omitted.

### Comparison of detection rate of overweight and obesity among adolescents' lifestyle patterns

It can be seen from Table 4 that there were significant differences in the detection rate of overall overweight and obesity among the adolescents with 6 lifestyle patterns (P < 0.01), in which, the detection rate of “high exercise-high calorie diet” class (class 6) was the lowest (15.9%), and the detection rate of “low consciousness-low physical activity” class (class 1) was the highest (20.9%). In the junior high school group, the “high exercise-high calorie diet” class (class 6) had the lowest detection rate of overweight and obesity (15.6%), and the “low consciousness-low physical activity” class (class 1) had the highest detection rate (22.0%). In contrast, in the senior high school group, the “low exercise-high calorie diet” class (class 3) had the lowest detection rate of overweight and obesity (14.9%), and the “low consciousness-low physical activity” class (class 1) had the highest detection rate (19.9%), all of the above detection rate were statistically significant (P < 0.01).

**Table 4:** Comparison of overweight and obesity detection rates of adolescents' lifestyle patterns.

**Gender**	**Class**	**Statistical value**	**Junior high school**		**Senior high school**		**Total**	
			**Number of people**	**Number of detected persons**	**Number of people**	**Number of detected persons**	**Number of people**	**Number of detected person**
Male	Class 1: Low consciousness-low physical activity class		399	75 (18.8)	414	93 (22.5)	813	168 (20.7)
	Class 2: Moderate exercise- balanced class		347	69 (19.9)	285	49 (17.2)	632	118 (18.7)
	Class 3: Low exercise-high calorie diet class		1279	221 (17.3)	1428	242 (16.9)	2707	463 (17.1)
	Class 4: Low consciousness-unhealthy class		490	94 (19.2)	502	99 (19.7)	992	193 (19.5)
	Class 5: Low diet behavior class		300	46 (15.3)	333	58 (17.4)	633	104 (16.4)
	Class 6: High exercise-high calorie diet class		646	94 (14.6)	488	84 (17.2)	1134	178 (15.7)
		Value of χ^2^		7.681		8.005		11.892
		Value of *P*		0.175		0.1565		0.036
Female	Class 1: Low consciousness-low physical activity class		454	113 (24.9)	560	101 (18.0)	1014	214 (21.1)
	Class 2: Moderate exercise- balanced class		206	41 (19.9)	149	19 (12.8)	355	60 (16.9)
	Class 3: Low exercise-high calorie diet class		1563	269 (17.2)	1662	217 (13.1)	3225	486 (15.1)
	Class 4: Low consciousness-unhealthy class		646	155 (24.0)	557	95 (17.1)	1203	250 (20.8)
	Class 5: Low diet behavior class		231	48 (20.8)	255	37 (14.5)	486	85 (17.5)
	Class 6: High exercise-high calorie diet class		266	48 (18.0)	210	30 (14.3)	476	78 (16.4)
		Value of χ^2^		21.530		11.497		31.897
		Value of *P*		0.001		0.042		0.000
Total	Class 1: Low consciousness-low physical activity class		853	188 (22.0)	974	194 (19.9)	1827	382 (20.9)
	Class 2: Moderate exercise- balanced class		553	110 (19.9)	434	68 (15.7)	987	178 (18.0)
	Class 3: Low exercise-high calorie diet class		2842	490 (17.2)	3090	459 (14.9)	5932	949 (16.0)
	Class 4: Low consciousness-unhealthy class		1136	249 (21.9)	1059	194 (18.3)	2195	443 (20.2)
	Class 5: Low diet behavior class		531	94 (17.7)	588	95 (16.2)	1119	189 (16.9)
	Class 6: High exercise-high calorie diet class		912	142 (15.6)	698	114 (16.3)	1610	256 (15.9)
		Value of χ^2^		24.760		17.193		38.175
		Value of *P*		0.000		0.004		0.000

The number in the brackets is the detection rate /%.

At the level of 6 lifestyle patterns of adolescents of different genders, there was only a statistically significant difference in the detection rate of overweight and obesity among male students (P < 0.05). The detection rate of “high exercise-high calorie diet” class (class 6) was the lowest (15.7%), and the detection rate of “low consciousness- low physical activity” class (class 1) was the highest (20.7%). The detection rate of overweight and obesity in the female students of junior high school group, senior high school group and overall group were statistically significant (P < 0.01). The lowest detection rates of overweight and obesity were “low exercise-high calorie diet” class (class 3) (17.2%), “moderate exercise-balanced” class (class 2) (12.8%) and “low exercise-high calorie diet” class (class 3) (15.1%), respectively. Alternatively, the highest detection rates of overweight and obesity were “low consciousness-low physical activity” class (class 1) (junior high school group: 24.9%; senior high school group: 18.0%; overall group: 21.1%).

### Logistic regression analysis on the influencing factors of overweight and obesity detection rate

As can be seen from Table 5, age group was the independent influencing factor of overweight and obesity among Chinese adolescents (*P* < 0.01), and the overweight and obesity probability of junior high school group was 1.16 times that of senior high school group (1/OR senior high school). Compared with the “high exercise-high calorie diet” class (class 6), the “low consciousness-low physical activity” class (class 1) and “low consciousness-unhealthy” class (class 4) were 1.432 times and 1.346 times higher than the “high exercise-high calorie diet” class (class 6), respectively.

**Table 5:** Multivariate logistic regression analysis of overweight and obesity.

**Independent variable**		***B***	**Standard error**	***OR***	***OR* value 95%CI**	***P***
Gender	Female			1.00		
	Male	0.041	0.046	1.042	0.952–1.140	0.370
Age group	Junior high school			1.00		
	Senior high school	−0.153	0.045	0.858	0.786–0.938	0.001
Regions	East			1.00		
	Middle	0.100	0.055	1.106	0.993–1.231	0.067
	West	−0.045	0.056	0.956	0.857–1.067	0.421
Classes	Class 1: High exercise-high calorie diet type			1.00		
	Class 2: Low consciousness-low physical activity type	0.359	0.090	1.432	1.200–1.708	0.000
	Class 3: Moderate exercise-balanced type	0.155	0.107	1.168	1.168–0.946	0.149
	Class 4: Low exercise-high calorie diet type	0.033	0.078	1.034	0.888–1.204	0.670
	Class 5: Low consciousness-unhealthy type	0.297	0.087	1.346	1.134–1.598	0.001
	Class 6: Low diet behavior type	0.089	0.105	1.093	0.889–1.343	0.400

The gender is based on girls, the age group is based on junior high school, and the region is based on the east; the lifestyle pattern is based on high exercise-high calorie diet type.

## Discussion

### Analysis of adolescents' lifestyle patterns

The results indicated that the adolescents' lifestyle patterns can be divided into the class of “low consciousness-low physical activity” class (class 1), “moderate exercise-balanced” class (class 2), “low exercise-high calorie diet” class (class 3), “low consciousness-unhealthy” class (class 4), “low diet behavior” class (class 5) and “high exercise-high calorie diet” class (class 6).

Among the above types, the adolescents with “low exercise-high calorie diet” class (class 3) accounted for the highest proportion. In the recent decades, tremendous changes had taken place in the aspects of economic development, social and cultural changes, food safety and built environment, resulting in significant changes in the lifestyle of adolescents (27, 28). The main manifestation focused on the continuous reduction of sleep duration, lack of interest in exercise, increase in irregular diet, as well as the more time spent on video (29). Adolescence is a key stage in the development of a healthy lifestyle, which is closely linked to its time and will develop along with the economic and social development. At present, the Internet has penetrated into the lives and studies of Chinese adolescents, bringing them convenience but also becoming an important factor affecting their health. The excessive use of the Internet and electronic devices has led to a reduction in outdoor activities and sleep time for young people, which in turn has led to metabolic disorders or disturbances in their biological clocks, and has even affected their psychological health, thus adversely affecting their healthy lifestyles (23). In addition, the behaviors such as unhealthy diet, low physical exercise would also affect the development of a variety of health-risk behaviors, and moreover, the factors including self-control level and family environment were also important predictors (30).

The results of the present study demonstrated that the males were inclined to enter the “high exercise-high calorie diet” class (class 6) and “moderate exercise-balanced” class (class 2). The previous results reported that the females were more prone to exhibit low exercise and sedentary behavior, as compared to males (18), which was consistent with the results of this study. The main reason causing females' physical exercise was usually less active than that of males lied in the gender difference of perception barriers, in which, lack of exercise energy and willpower were listed as the main factors (31). Besides, it was also influenced by the phenomenon of gender inequality (32, 33). The results also illustrated that the junior high school group was less likely to enter the class of “low consciousness-low physical exercise” (class 1), “low exercise-high calorie” (class 3), “low consciousness-unhealthy” (class 4) and “low diet behavior” (class 5). This may be attributed to the fact that junior high school students were constrained and regulated by their families and schools in the aspects of daily routine, eating habits, physical exercises, etc., which has also been confirmed by Ji Gang et al. (34). On the other hand, with the increase of age, adolescents' autonomy and the interaction with their peers also enhanced, which would exert an impact on adolescents' lifestyle (35).

### Analysis on the clustering characteristics of adolescents' lifestyle

The results indicated that in the clustering of various factors of adolescents' lifestyle, the clustering of low physical exercise and high intake of snacks and carbonated beverages was more common. The clustering of these health-risk behaviors may be related to a series of variations in individual, social and environmental factors induced by China's rapid economic development in recent decades.

Under the influence of various factors such as educational needs (36), Chinese teenagers have been overburdened in their studies and spent less time in extracurricular activities. Moreover, physical education was mainly conducted based on the principle of safety, it was difficult to achieve effective exercise loads and frequency in physical education, and leading limited effect of cultivating students' physical exercise habits (37). Furthermore, due to the influence of family economic level, parents' support attitude, and surrounding sports resources, Chinese adolescents had a relatively low probability to participate in paid sports activities (38, 39). Besides, the natural environment such as air pollution and noise pollution, as well as the built environment such as the suitability of physical exercise in the community, would affect the participation of adolescents in physical exercise (40). These above mentioned factors would affect the training of adolescents' interest in exercise, skill mastery, exercise motivation and psychological simulation, and thereby limiting the subjective initiative and spontaneity of the participation in physical exercise.

According to the survey of the China Health and Nutrition Survey (CHNS) project conducted by the China Center for Disease Control and Prevention and the Population Center of the University of North Carolina in the United States, the proportion of unhealthy snack consumers with high salt, high fat and high energy among Chinese children and adolescents presented a upward trend (41), because of the increasing number of high-income families and economically developed regions. With the improvement of the economic level, the dietary consumption behavior and motivation of the teenagers have transformed. People began to pursue experience and satisfaction in delicious food, instead of just having warm clothing and adequate food (42). Meanwhile, under the influence of more and more advertisements concerning high-calorie and low-nutrition foods, the adolescents tended to consume such kind of foods. On the other hand, it could be found from the 2015 survey data of the Chinese General Social Surver (CGSS), the phenomenon of grandparents upbringing existed in 38.35% of Chinese adolescents. It should be noted that the health literacy of middle-aged and elderly groups were obviously lower, and the generation gap and the transfer of consumption rights would aggravate the consumption behavior of unhealthy diets among adolescents (43).

### Relationship between overweight and obesity and lifestyle patterns in adolescents

In the comparison of lifestyle patterns of overweight and obesity, it was found that the detection rate of overweight and obesity among adolescents with “high exercise-high calorie diet” class (class 6) was the lowest. In contrast, the risk of overweight and obesity prominently increased in the “low consciousness-low physical activity” class (class 1) and the “low consciousness-unhealthy” class (class 4).

Although the adolescents with “high exercise-high calorie diet” class (class 6) had higher intake of snacks and carbonated beverages, the detection rate of overweight and obesity was still the lowest. For the adolescents in the growing stage, higher levels of physical exercise could effectively facilitate energy metabolism and offset the risk of nutritional status caused by high calorie intake. Moreover, as an important approach to prevent and intervene overweight and obesity, physical exercise could not only reduce the biochemical markers of obesity, but also contribute to individual's mental health (44) and self-regulation (45). To a certain extent, good mental health, emotion/behavior, and social adaptation could also contribute to a healthier nutritional status (46–48). Moreover, organized physical exercise could promote more healthy behaviors in adolescents (49), and thus exhibiting more positive performance on the individual's nutritional status. Similar results could be found in the present study, with “high exercise-high calorie diet” class (class 6) adolescents trending toward positive health behaviors in both health consciousness and sleep duration. As a result, regular physical exercise is conducive to building a positive environment and lifestyle for teenagers.

Compared with the “high exercise-high calorie diet” class (class 6), the “low consciousness-low physical activity” class (class 1) and “low consciousness-unhealthy” class (class 4) adolescents had a higher risk of overweight and obesity. It has been reported that health consciousness could help adolescents identify positive healthy behaviors in their lifestyles, thereby helping them change health-risk behaviors to maintain a good living condition, which would be beneficial to effectively promote individuals to live in a healthy nutritional status (50). The following conclusions could be drawn after integrating various personal health behavior variation theories: health consciousness may affect the distal consciousness of behaviors through attitude and self-efficacy, and served as an important factor in the pre-motivation stage (51). Therefore, it can be deduced that low health consciousness could lead to a series of health-risk behaviors such as low physical exercise and poor diet, thereby increasing the risk of overweight and obesity. Additionally, a certain lifestyle of adolescents may trigger the development of a variety of health-risk behaviors (52), increase the interaction performance of health-risk behaviors, and thus elevating the hazard of overweight and obesity.

In this study it was also found that health risk behaviors among adolescents did not occur in isolation, but rather a mixture of healthy and unhealthy behaviors. Evidence suggests that adolescents' lifestyles are often combined with non-modifiable and modifiable covariates into different lifestyle patterns (53). One explanation may include the theory of problem behavior, which suggests that an underlying behavioral syndrome causes adolescents to engage in multiple problem behaviors, possibly caused by an imbalance of risk factors in relation to protective factors in the personality and socio environmental domains (54). Longitudinal studies demonstrated that screen time could, to a large extent, drive adolescents to produce behaviors including poor diet, reduced physical activity and sleep disorders, resulting in an increased risk of obesity (55, 56). Nevertheless, lifestyle patterns in this study tended to be positive healthy behaviors in video time, which may be correlated with the influence of family and school constraints and academic pressure from Chinese middle school. At present, there are few studies on the relationship between lifestyle patterns and individual invariant covariates and their changing trends, and the direct relationship between health behaviors remains to be clarified.

In conclusion, the clustering types of adolescents' lifestyles are relatively complicated. In the future, more characteristics of the investigators, including mental health, whether they live in school, and family environment, should be considered to better explain the potential confounding factors. Considering that the cumulative effect of adolescents' lifestyles on health is relatively sophisticated, in-depth research on the development and changes of adolescents' lifestyle patterns should be further investigated, so as to more scientifically explore the relationship between lifestyles and overweight and obesity.

## Data availability statement

The raw data supporting the conclusions of this article will be made available by the authors, without undue reservation.

## Ethics statement

Ethical review and approval was not required for the study on human participants in accordance with the local legislation and institutional requirements. Written informed consent to participate in this study was provided by the participants' legal guardian/next of kin.

## Author contributions

Conceptualization: YM and HW. Methodology: JS. Software: JinW. Validation: JiaW, JS, and YH. Formal analysis and writing—original draft preparation: YM. Investigation: HW, JinW, and YH. Resources, supervision, project administration, and funding acquisition: HW. Data curation: YM and JiaW. Writing—review and editing: HW, JS, and YH. Visualization: JiaW and JinW. All authors have read and agreed to the published version of the manuscript.

## Funding

This study was funded by the Ministry of Education Youth Fund Project (granted: 21YJC890026) and Shanxi Province Philosophy and Social Sciences Planning Project (granted: 2021YJ121).

## Conflict of interest

The authors declare that the research was conducted in the absence of any commercial or financial relationships that could be construed as a potential conflict of interest.

## Publisher's note

All claims expressed in this article are solely those of the authors and do not necessarily represent those of their affiliated organizations, or those of the publisher, the editors and the reviewers. Any product that may be evaluated in this article, or claim that may be made by its manufacturer, is not guaranteed or endorsed by the publisher.
